# What is the best surgical approach for esophageal cancer?

**DOI:** 10.1515/iss-2023-0021

**Published:** 2024-12-06

**Authors:** Verena Tripke, Vladimir J. Lozanovski, Carolina Mann, Hauke Lang, Peter P. Grimminger

**Affiliations:** Department of General, Visceral and Transplantation Surgery, University Medical Center of the Johannes Gutenberg University Mainz, Mainz, Germany

**Keywords:** esophagectomy, minimally invasive techniques, robotic assisted approach, laparoscopic/thoracoscopic approach

## Abstract

Esophageal cancer is an aggressive tumor entity, and oncologic esophagectomy with two-field lymphadenectomy after perioperative chemotherapy or chemoradiotherapy is the standard of care for curative treatment. Oncological esophagectomy is a complex procedure associated with a relevant surgical trauma. Complications, such as severe pulmonary infections and anastomotic leakage with mediastinitis lead to a high morbidity rate. To reduce the surgical trauma, the minimally invasive technique was introduced in esophageal surgery. Minimally invasive esophagectomy is associated with less postoperative pain and a reduced rate of pulmonary infections. Currently, there are two major different totally minimally invasive techniques, the conventional laparoscopic/thoracoscopic approach (MIE) and the robotic assisted approach (RAMIE). Both methods require teaching due to the flat learning curve associated with these complex procedures. However, both MIE and RAMIE are performed safely in specialized centers. They are associated with improved short-term outcome and similar oncological outcome compared to open esophagectomy. The robotic assisted approach has additional benefits that may be supported by the results of more randomized controlled trials in the future.

## Introduction

Esophageal cancer is an aggressive malignancy, with approximately 600,000 new cases worldwide per year and 540,000 deaths globally in 2020 [1]. Today, the standard of care for curative treatment of esophageal cancer is a multimodal therapy consisting of chemotherapy or chemoradiotherapy and oncological esophagectomy [2], 3]. The 5-year survival rate after surgery is 40 %, compared to 10 % for those treated without surgery [4]. The standard surgical procedure for patients with esophageal cancer or cancer of the gastroesophageal junction (GEJ) includes transthoracic esophagectomy with two-field lymphadenectomy, gastric pull up, and intrathoracic (Ivor-Lewis) or cervical (McKeown) anastomosis. Regarding the subgroup GEJ type Siewert II, transhiatal extended gastrectomy is still performed in many centers, assuming benefits for postoperative quality of life when avoiding thoracic access. Whether it equals the transthoracic approach in terms of oncological outcome is currently being investigated in the CARDIA trial.

However, oncological transthoracic esophagectomy is a technically challenging and complex procedure, associated with significant surgical trauma by involving at least two body cavities. Due to technical and medical innovations, as well as improved complication management the mortality rate for oncological esophagectomy has been reduced to less than 5 %. Half of the patients, who received open esophagectomy still suffered from cardiopulmonary complications [5], which are associated with a longer stay in intensive care units, a longer hospital stay, and significant deterioration of quality of life. The minimally invasive technique avoids simultaneous laparotomy and thoracotomy. The first series of five cases in 1992 were hybrid minimally invasive esophagectomies, combining camera-assisted thoracoscopy with laparotomy [6]. They were followed by case series reporting on hybrid esophagectomies, including thoracotomy and laparoscopy, or totally minimally invasive esophagectomies, including thoracoscopy and laparoscopy [7]. However, the minimally invasive approach is technically even more challenging and requires both laparoscopic und thoracoscopic skills. The thoracic part of the operation is particularly demanding, as the esophagus is here located closely to sensitive structures. Furthermore, concerns have arisen about whether the minimally invasive procedure provides equal oncological results.

Until now, there are two different totally minimally invasive techniques: the conventional laparoscopic/thoracoscopic approach and the robotic-assisted approach. The robotic-assisted minimally invasive esophagectomy (RAMIE) was introduced in the early 2000s. In 2006, the first series of 21 robot-assisted thoracoscopic esophagectomies was published by van Hillegersberg et al. [8]. The robotic-assisted approach benefits from a three-dimensional view and articulated instruments with 7 degrees of freedom and allows a more precise dissection, especially in the thoracic part of the operation.

## Minimal invasive or open esophagectomy?

The first large series of totally minimally invasive esophagectomy (MIE) was published in 2003, with 222 cases [9]. This study demonstrated that MIE was technically feasible and safe, resulting in a median hospital stay of 7 days (range, 3–75) and a low operative mortality of 1.4 % (n=3). In 2012 Luketich et al. published a series of 1,033 consecutive patients undergoing MIE [10]. Primary endpoint of this analysis was 30-day mortality. Additionally, the results of performing the anastomosis in the neck (McKeown) and the intrathoracic anastomosis (Ivor–Lewis) were compared. The Ivor–Lewis MIE featured significantly reduced rate of laryngeal nerve injury (1–8 %, p<0.001) and low mortality rate of 0.9 %. The authors consider Ivor–Lewis MIE as their favored approach. To create further evidence for the use of minimal invasive esophagectomy, randomized controlled trials were conducted.

The randomized controlled MIRO trial compared hybrid esophagectomy (HMIE), which consists of a conventional laparoscopic abdominal part and an open thoracic part, to open transthoracic esophagectomy (OE). The HMIE group showed a lower incidence of intraoperative and postoperative major complications compared to the OE group (36 vs. 64 %, p<0.001) [11].

Another randomized controlled trial comparing HMIE to OE was the MIOMIE trial, published in 2018 [12]. The trail was stopped after including 26 patients due to a high rate of anastomotic leakages. However, there was no difference in short-term and long-term oncological results between the two groups. In 2012, Biere et al. published a multicenter randomized controlled trail that compared open esophagectomy to conventional laparoscopic/thoracoscopic esophagectomy (MIE), known as the TIME-trial [13]. Here, only 9 % of the patients in MIE group had pulmonary infections in the first two weeks after the operation compared to 29 % in the OE group (p=0.005). The in-hospital mortality was similar. MIE was also superior to OE regarding length of hospital stay, postoperative pain, and quality of life. The long-term oncological outcomes of the study were published in 2017 and were comparable between the two groups [14]. Another multicenter randomized controlled trial comparing open to minimal invasive esophagectomy is the ROMIO trial from the UK [15]. OE and HMIE will be compared with recovery of physical function up to three months as the primary outcome. The ROMIO-trial includes also a randomized substudy to generate data on MIE and to compare MIE to HMIE. It may demonstrate benefits from MIE over HMIE.

Many meta-analyses and retrospective data analyses support the benefits of MIE as well. A meta-analysis from 2016 reviewed thirteen studies including 1,549 patients [16]. Patients who underwent MIE had a significant lower rate of complications and less intraoperative blood loss. The oncological long-term results showed no difference between the two groups. A propensity-score matched analysis from 2018 compared MIE and HMIE, demonstrating less postoperative pain, shorter ICU stay, and a lower rate of postoperative pulmonary infections. The incidence of overall postoperative complications was equal between the two groups [17].

Benchmarks for MIE in low-risk patients (<66 years, body mass index 19–29 kg/m^2^, ECOG score ≤1, American Society of Anesthesiologists score ≤2) were defined in 2017 and included 1,057 MIEs [18]. Ivor–Lewis esophagectomy with high intrathoracic anastomosis was performed in 56.3 % of the cases, and McKeown esophagectomy with cervical anastomosis in 43.7 % of the cases. The median hospital stay was 12 days and 26.9 % of the patients developed major complications ≥ grade III according to Clavien–Dindo classification [19]. Benchmarks for 30- and 90-day mortality were ≤1 % and ≤4.6 %, respectively.

## Robotic-assisted esophagectomy

To overcome limiting factors in MIE, such as rigid instruments and limited movement, robotic assistance found its way to esophageal surgery. After introducing robotic-assisted esophagectomy in 2003, the number of publications demonstrating the feasibility and safety of RAMIE has been constantly rising [20], [21], [22], [23]. The results of the Multicenter International Registry from twenty centers in Europe, Asia, North and South America were published in 2022. In total, 856 patients underwent RAMIE between 2016 and 2019. In 73 % of the cases, an Ivor-Lewis esophagectomy, and in 27 % the McKeown approach was used [24]. To achieve further evidence for RAMIE, the single-center randomized controlled ROBOT trial was conducted [25]. This trial compared RAMIE to OE including 112 patients. RAMIE was superior to OE regarding overall surgery-related postoperative complications (59 vs. 80 %, p=0.02). Patients in RAMIE group had significant less median blood loss, lower incidence of postoperative cardiopulmonary complications, less postoperative pain, and a better quality of life. The short- and long-term oncological outcomes were similar between RAMIE and OE. However, is RAMIE superior to MIE? The 2022 published RAMIE trial was the first randomized trial comparing conventional minimally invasive esophagectomy with the robotic approach in patients with esophageal squamous cell carcinoma [26]. This study showed a shorter operation time (203.8 vs. 244.9 min, p<0.001). There was no difference in postoperative complications and 90-day mortality, but RAMIE showed an improved efficiency of thoracic lymph node dissection and a higher rate of lymph node dissection along the left laryngeal nerve (79.5 vs. 67.6 %, p=0.001). For improved overall survival, a high lymph node yield with at least 15 resected nodes has proven to be crucial [27], 28]. Until now, only the short-term outcomes were published, and the follow-up data of the study are still lacking. Another randomized controlled trial developed to compare RAMIE to MIE in patients with esophageal squamous cell carcinoma is the REVATE-trial [29]. The primary endpoint is the success rate of left recurrent laryngeal nerve lymph node dissection. Success was defined as the removal of at least one lymph node without causing nerve damage lasting longer than 6 months. Secondary endpoints were postoperative recovery, length of hospital-stay, 30- and 90-day mortality, quality of life, and oncological outcome. Recruiting started in November 2018 and ended in March 2022. In total, 203 patients from three Asian centers were included in the analysis. The rate of successful left recurrent laryngeal nerve lymph node dissection was higher in the robotic group (88.3 vs. 69 %, p<0.001). One week after surgery, the robotic group had a lower incidence of left recurrent laryngeal nerve palsy compared to MIE group (20.4 vs. 34 %, p=0.029), and the permanent recurrent laryngeal nerve palsy rates at 6 months were 5.8 and 20 % (p=0.003). Furthermore, more mediastinal lymph nodes were resected in the robotic group (p=0.035). In conclusion, the robotic approach leads to a higher success rate left recurrent laryngeal nerve lymph node dissection with a lower rate of recurrent laryngeal nerve injury. Especially in patients with esophageal squamous cell carcinoma, a precise and radical dissection of the recurrent laryngeal lymph nodes is important as lymph node metastasis is the most important prognostic factor, and the robotic approach seems to facilitate the dissection easier in these patients.

The multicenter randomized controlled ROBOT2 trial started in January 2021 in Europe and compares MIE to RAMIE in patients with esophageal adenocarcinoma [30]. In total, 218 patients will be recruited and randomized to RAMIE or MIE group. The primary outcome is not the short-term outcomes, as both techniques are minimally invasive, but the number of resected abdominal and mediastinal lymph nodes specified per lymph node station. The results of the last two studies mentioned above might provide further evidence for the benefits of RAMIE over MIE and are awaited with great interest.

## Conclusions

Oncological esophagectomy is the cornerstone in curative treatment for patients with esophageal cancer or cancer of the gastroesophageal junction. Both totally minimally invasive techniques for esophagectomy, MIE and RAMIE, are routinely used in specialized centers for curative treatment of esophageal cancer. However, minimally invasive esophagectomy is a complex procedure and is associated with a flat learning curve. Minimally invasive esophagectomy shows better short-term outcomes for patients with esophageal cancer compared to the open approach. Several randomized controlled trials revealed significantly fewer pulmonary infections and less postoperative pain in the minimally invasive group. Both Ivor-Lewis and McKeown esophagectomy are performed safely using minimally invasive techniques. Furthermore, the minimally invasive approach delivers equal long-term oncological results compared to open esophagectomy. The robotic approach, due to its three-dimensional view and articulated instruments with seven degrees of freedom, may offer additional benefits in preservation of the laryngeal nerve and a higher rate of resected lymph nodes. The 2022 RAMIE trial reported a higher rate of lymph node dissection along the left laryngeal nerve and improved efficiency of thoracic lymph node dissection. Especially in patients with esophageal squamous cancer with high tumor localization, the robotic approach might make the dissection easier. However, for definite conclusions, more results from randomized controlled trails are needed. The ROBOT2 trial may provide new results to confirm the superiority of RAMIE over MIE.
